# Heterogeneity and chronology of 6q15 deletion and ERG-fusion in prostate cancer

**DOI:** 10.18632/oncotarget.6597

**Published:** 2015-12-14

**Authors:** Martina Kluth, David Meyer, Antje Krohn, Fabian Freudenthaler, Melanie Bauer, Georg Salomon, Hans Heinzer, Uwe Michl, Stefan Steurer, Ronald Simon, Guido Sauter, Thorsten Schlomm, Sarah Minner

**Affiliations:** ^1^ Institute of Pathology, University Medical Center Hamburg-Eppendorf, Hamburg, Germany; ^2^ Prostate Cancer Center, Martini-Clinic, Hamburg, Germany; ^3^ Department of Urology, Section for Translational Prostate Cancer Research at University Medical Center Hamburg-Eppendorf, Hamburg, Germany

**Keywords:** 6q deletion, prostate cancer, ERG, MAP3K7, heterogeneity

## Abstract

Prostate cancer is notorious for its heterogeneity, which poses a problem for the applicability of diagnostic molecular markers. However, heterogeneity analysis can provide valuable information on the chronology in which molecular alterations arise. Here, we constructed a heterogeneity tissue microarray (TMA) comprising samples from 10 different tumor areas of 189 prostate cancers each in order to study the sequence of two frequent molecular alterations, i.e. 6q15 deletion and *TMPRSS2:ERG* fusion. Previous work shows a marked inverse relationship between these alterations, suggesting that presence of one of these alterations might impact development of the other. 6q15 deletion was analyzed by fluorescence *in situ* hybridization and ERG-expression by immunohistochemistry. Only 6.6% of 334 ERG-positive but 28.4% of 440 ERG-negative TMA spots showed 6q15 deletions (*p* < 0.0001). A breakdown of these data to the level of tumor foci revealed 6q deletions in 138 tumor foci that were large enough to have at least 3 analyzable TMA spots. These included 42 tumor foci with homogeneous ERG positivity and 16 with homogeneous 6q15 deletions. Remarkably, six of the 42 homogeneously *ERG*-positive tumor foci (14.3%) harbored small 6q15-deleted areas, but none of the 34 6q15-deleted foci showed areas of ERG positivity (*p* = 0.022). In conclusion, our data suggest that *ERG*-fusion can precede 6q15 deletion, but not vice versa. The complete absence of ERG-positive tumor areas in 6q15-deleted tumor foci further suggest that the functional consequences of 6q15 deletions may prevent the development of *TMPRSS2:ERG* fusions.

## INTRODUCTION

As prostate cancer is already notorious for substantial heterogeneity on the morphologic level, it is conceivable, that substantial heterogeneity may also occur for molecular features. On the one hand heterogeneity may limit the applicability of diagnostic, prognostic, and predictive molecular markers, but, on the other hand, it may help to understand the chronology of molecular events during tumor progression.

Studies by others and us have demonstrated, that the *TMPRSS2*:*ERG* fusion status is highly heterogeneous within individual prostate cancers [1–3]. Deletions on chromosome 6q are the second most common deletions in prostate cancer and occur in 18–62% [4–9]. Data suggest a minimally deleted area on 6q15 with a length of 3–4 Mb containing only few genes, including *MAP3K7*/*TAK1*. A tumor-suppressive role in prostate cancer cells has recently been demonstrated for this gene [10]. The TGF-β and BMP activated kinase MAP3K7 is essential for a series of cancer associated signaling pathways like the p38 and JNK [11–13]. In a recent study, we found a strong association of *MAP3K7* deletion with early PSA recurrence in a series of 2,289 cancers analyzed by FISH. Remarkably, there was also a striking inverse relationship between *MAP3K7* deletions and presence of *TMPRSS2*:*ERG* fusions [9]. This finding raises the question whether *MAP3K7* deletion prevents cancers cells from developing *TMPRSS2*:*ERG* fusions or vice versa.

To thoroughly evaluate the heterogeneity for *MAP3K7* deletions and *TMPRSS2*:*ERG* fusion status and their interrelationship in prostate cancer, we took advantage of a newly developed heterogeneity tissue microarray (TMA) containing samples from 10 different tumor blocks of 189 large prostate cancers. This approach enables a high-throughput mapping of molecular features across entire tumors. The data show substantial heterogeneity for *MAP3K7* deletion in prostate cancer and suggest that *MAP3K7* deletion largely prevents development of *TMPRSS2*:*ERG* fusions.

## RESULTS

### Heterogeneity of *MAP3K7* deletion in prostate cancer

**Figure 1:**
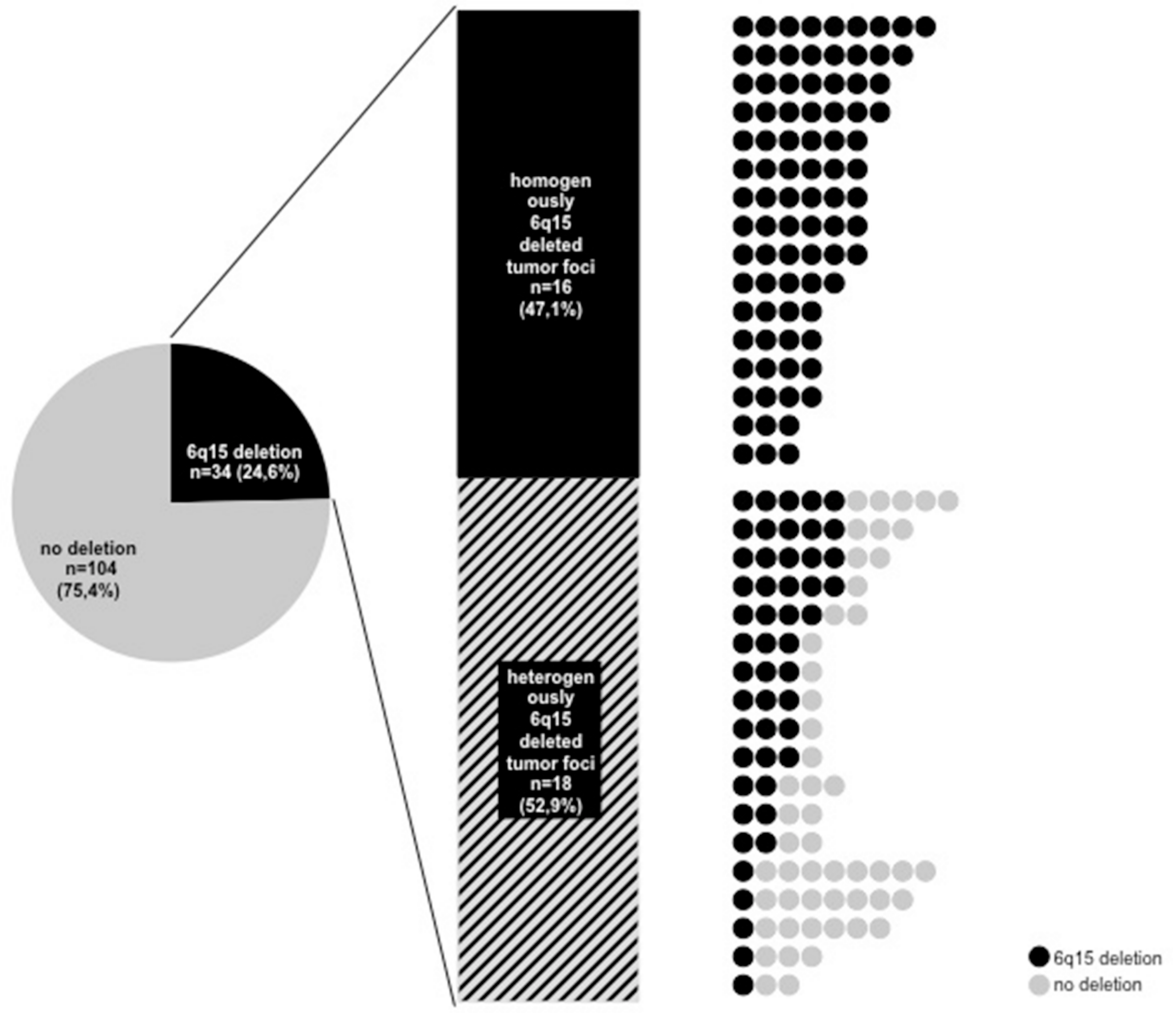
Heterogeneity of 6q15 deletion in all tumor foci with at least three analyzable tumor spots Right: All circles in one line corresponds to one tumor foci, black circle: 6q15 deleted tumor spot, grey circle: 6q15 non-deleted tumor spot.

The 6q15 FISH analysis of our heterogeneity TMA revealed 924 informative cancer spots (48.9% of 1,890 samples arrayed). 966 of 1,890 tissue spots were non-informative due to lack of tissue samples, absence of unequivocal cancer tissue or to lacking FISH signals for centromere 6, *MAP3K7* or both. A *MAP3K7* deletion was found in 165 (17.8%) of 924 cancers spots. All of these deletions were heterozygous and 140 patients had at least three informative cancer spots. Of these, 15 were homogeneously and 21 were heterogeneously *MAP3K7* deleted. 104 of 140 patients showed no *MAP3K7* deletion. To exclude interfocal tumor heterogeneity in patients with more than one tumor focus, we analyzed individual cancer foci for heterogeneity. There were 133 patients (95.0%) with 138 tumor foci large enough to create 3 informative cancer-containing tissue spots. Of these 138 tumor foci, there were 16 with homogeneous 6q15 deletion, 18 with heterogeneous 6q15 deletion, and 104 with a normal 6q15 result in all spots (Figure 1).

### *MAP3K7* deletion vs. ERG-expression

**Figure 2:**
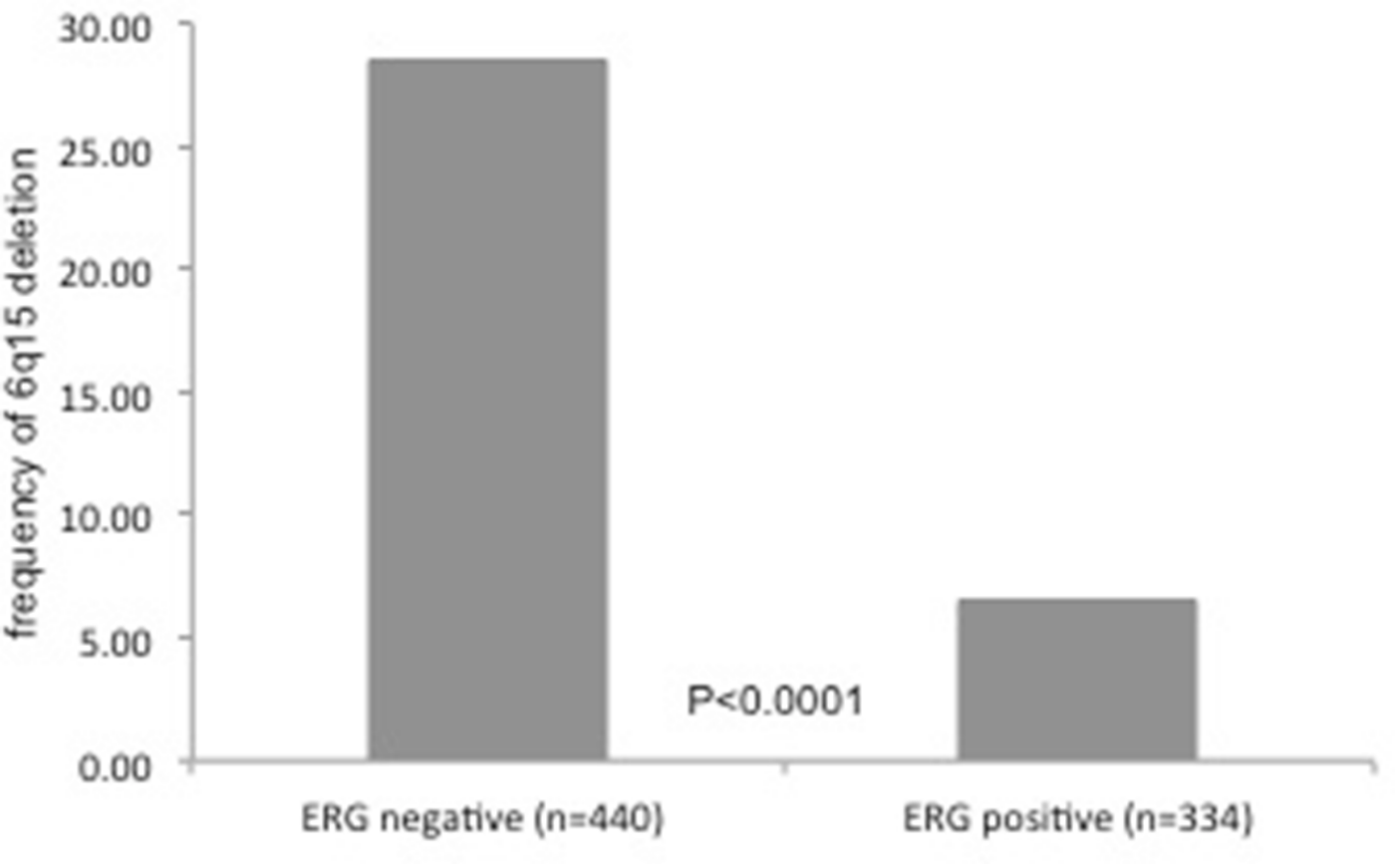
Relationship between *MAP3K7* (6q15) deletion status and ERG-fusion status measured by immunohistochemistry

ERG data were available from a previous study in 774 of 924 tissue spots with informative 6q15 deletion status. Positive ERG status was strongly linked to absence of 6q15 deletion in these samples. 6q15 deletion was seen in 125 (28.4%) of 440 ERG-negative, but only in 22 (6.6%) of 334 ERG-positive samples (*p* < 0.0001, Figure 2). The evaluation of the above mentioned 138 tumor foci revealed 42 tumor foci with homogeneous and 38 tumor foci with heterogeneous ERG positivity, while 56 tumor foci were ERG-negative.

### Sequel of *MAP3K7* deletion and *TMPRSS2*:*ERG* fusion development

**Figure 3:**
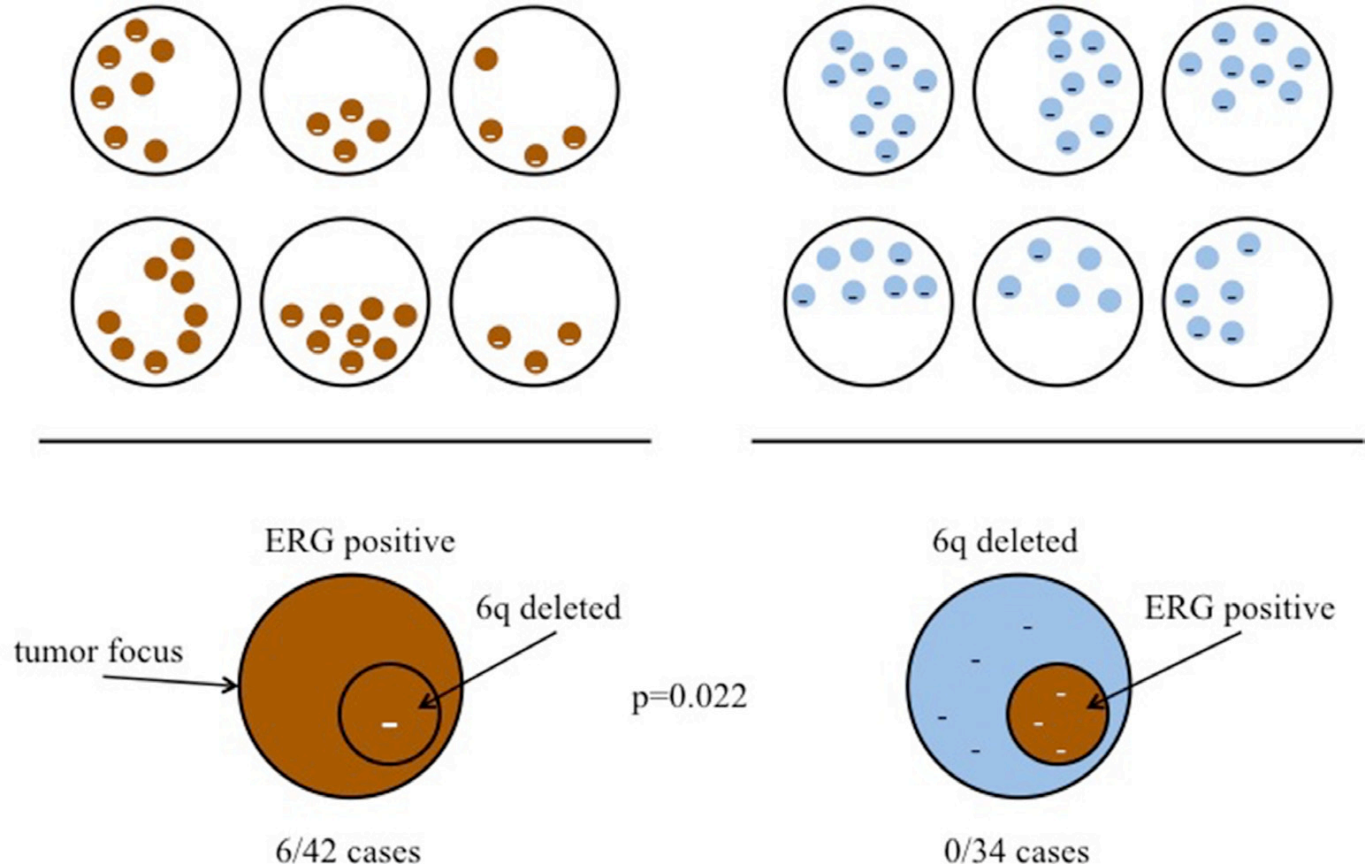
Sequel of *MAP3K7* (6q15) deletion and *TMPRSS2:ERG* fusion development Left: Cases with ERG-positive tumor focus and focal 6q15 deletion. Right: Cases with 6q15 deleted tumor focus and focal ERG positivity in the same tumor area.

To evaluate the chronological development of *TMPRSS2*:*ERG* fusion and 6q15 deletions we searched for tumors with focal 6q15 deletions arising in homogeneously ERG-positive cancers and tumors with focal ERG positivity arising in homogeneously or heterogeneously 6q15 deleted cancers. None of 34 cancers revealed focal ERG positivity in a 6q15 deleted setting, while 6 of 42 cancers had a small area of 6q15 deletion in an ERG-positive background (*p* = 0.022, Figure 3). Comparison of homogenously 6q15 deleted cancers lacking focal ERG positivity (*n* = 16) and homogenously ERG-positive cancers with focal 6q15 deletion (*n* = 6 of 42) reaches no statistical significance (*p* = 0.129, data not shown) most likely due to the small number of cases with homogenous alterations.

## DISCUSSION

Our data revealed that *MAP3K7* deletion is often heterogeneous in prostate cancer and prevents *TMPRSS2*:*ERG* fusion, while *TMPRSS2*:*ERG* fusion allows *MAP3K7* deletion.

To thoroughly evaluate the heterogeneity for *MAP3K7* deletions and *TMPRSS2*:*ERG* fusion status and their interrelationship in prostate cancer, we took advantage of a newly developed heterogeneity tissue microarray (TMA) containing samples from 10 different tumor blocks of 189 large prostate cancers. This approach enables a high-throughput mapping of molecular features across entire tumors.

The frequency of 25.7% *MAP3K7* deletions observed by FISH is somewhat higher than in our previous study describing 18.5% *MAP3K7* deletions on a TMA containing 2,289 different prostate cancers [9]. This is likely to be caused by the analysis of multiple samples per tumor in this study, enabling the identification of higher number of heterogeneous 6q15 deletions. Earlier studies using CGH, PCR or LOH analysis reported between 13% and 62% 6q15 deletion in prostate cancers [4–8, 14, 15]. Only one earlier study also used FISH with a probe specific for *MAP3K7* and found a deletion in 32% of 95 advanced stage tumors. As 6q15 deletions increase substantially with grade and stage, selection bias may have led to this slightly higher figure [7]. FISH represents the gold standard for the analysis of gene copy number changes. FISH enables a cell by cell analysis making the method independent of a possible pollution by non-neoplastic cells and genomic cancer heterogeneity. To fully exploit the strength of the FISH approach, stringent criteria were applied for assuring presence of cancer cells in each analyzed tissue spot and also for defining *MAP3K7* deletions. In an earlier study we had successfully validated our cut-off level of ≥ 60% deleted cell nuclei by comparing *PTEN* deletions detected by FISH and CGH arrays and demonstrating an 100% concordance [16].

Accumulating evidence suggests a critically important role of 6q15 deletions in prostate cancer. With a frequency of 20–30% observed in FISH studies, 6q15 deletions are among the most frequent genomic alterations in prostate cancer. The strong inverse association with *TMPRSS2*:*ERG* fusions identifies 6q15 deleted cancers as a significant subgroup among “non fusion-type” prostate cancers [8, 9]. Moreover, the strong association of these deletions with early PSA recurrence potentially assigns clinical relevance to this molecular subgroup [9], which, however, may be limited if the analyzed biomarker is only present in a fraction of a tumor.

Considering its importance, the number of studies systematically analyzing target heterogeneity in cancer is relatively small. Even studies addressing the issue of tumor heterogeneity often limit themselves to the analysis of one slide/block per tumor/patient. However, one tissue section may not completely represent the biology of a large cancer. “Heterogeneity TMAs” were thus manufactured by our group as new tools for studying molecular cancer heterogeneity [1, 17]. A TMA analysis of one sample each from ten different tumor-containing blocks distributed across the entire tumor enables a comprehensive three-dimensional high-throughput analysis of molecular features in large series of tumors. This heterogeneity TMA concept differs markedly from previous attempts to increase the representativity of prostate cancer TMAs, namely by sampling multiple cores from just one tumor block [18, 19]. The data of our study demonstrate that heterogeneity of 6q15 deletion occurs in about half of 6q15 deleted prostate cancers. This clearly limits the potential of treatment decisions based on the 6q15 deletion status determined on one single cancer biopsy.

While cancer heterogeneity impairs the applicability of diagnostic, prognostic, and predictive molecular markers, it can be utilized to understand the sequel of two or more molecular features occurring in one cancer. Depending on the sequel of development of *MAP3K7* deletions and ERG-expression, one can expect a small area of cancer having both alterations within a larger area having only the first one of these changes. Our observation that 6 of 42 homogeneously ERG-positive cancer foci had focal 6q15 deletions strongly suggests, that presence of *TMPRSS2*:*ERG* fusions does not prevent the development of 6q15 deletions. This is all the more the case as the frequency of 6q15 deletions (14.3%) in this subgroup did not differ significantly from the 6q15 deletion rate in the entire (unselected) cohort (36/140; 25.7%; *p* > 0.1). The complete absence of focal ERG positivity in 34 cancer foci with 6q15 deletions strongly supports the concept of 6q15 deletions either directly or indirectly preventing tumor cells from developing *TMPRSS2*:*ERG* fusions.

*TMPRSS2*:*ERG* fusion, and other rearrangements involving ETS family members, is facilitated through androgen-mediated activation of transcriptional programs generating inter- or intra-chromosomal ‘transcriptional hubs’ [20]. Androgen-induced, DNA double-strand breaks followed by low-fidelity DNA repair can then lead to genome alterations involving androgen-regulated genes (such as *TMPRSS2*), which can be fused to transcription factors with oncogenic activity (such as *ERG*) [21, 22]. It is well known, that the likelihood of such rearrangement events can be impacted by an altered function of chromatin modifying genes. The results of whole genome sequencing studies have highlighted a pivotal role of various chromatin regulating genes in prostate cancer [5, 23–25]. In an earlier study, we had demonstrated a striking association of 5q21 deletions with absence of *TMPRSS2*:*ERG* fusions and implicated *CHD1*, a chromatin modifying gene, as the target gene of this deletion. We were able to show that a reduced expression of CHD1 prevents the development of *TMPRSS2*:*ERG* fusions [26]. Also the 6q21–q22 region contains several genes involved in histone and chromatin modification, such as ASF1A, BEND3 and HDAC2. Of note, HDAC2 interacts with Topoisomerase IIB (TOP2B) in a protein complex that is essential for *ERG*-fusion development [27, 28]. It could, thus, be speculated, that the reduced expression of one or several genes involved in 6q deletion could affect the likelihood of *TMPRSS2:ERG* fusion development.

In summary, the results of this study show that *MAP3K7* deletion is often heterogeneous in prostate cancer. The data also suggest that presence of a 6q15 deletion including *MAP3K7* - either directly or indirectly - prevents tumor cells from developing *TMPRSS2*:*ERG* fusions.

## MATERIALS AND METHODS

### Patient samples and tissue microarray (TMA) construction

A total of 189 prostate cancers were used for manufacturing a prostate cancer heterogeneity TMA, as previously described [1]. All patients underwent radical prostatectomy at the Martini Clinic, Prostate Cancer Center, University Medical Center Hamburg-Eppendorf according to our institutional standard.

In brief, all prostates were prepared in a standardized way [29, 30]. Macroscopic images were taken from each tumor. Prostate cancer patients were selected for this study, if they had a tumor involvement of at least 10 different tissue blocks. These cancers had an average volume of 3.4 cm^3^ (maximum 60 cm^3^). For each cancer, the number of independent tumor foci was determined according to Wise et al. [31]. In brief, tumor areas were defined as part of a single focus if they were within 3 mm of each other in any section or within 4 mm on adjacent sections. This method identified 1–6 independent tumor foci in our prostate cancers. Seventy-six prostates had one tumor focus, 48 prostates had 2 tumor foci, 28 prostates had 3 tumor foci, and 38 prostates had 4 or more tumor foci. The latter group also included 10 prostates that contained multiple small and very small tumor foci rather than one or several clearly distinguishable tumor masses. From each of our 189 tumors ten different tumor containing tissue blocks were selected for TMA manufacturing. If more than 10 blocks were available, blocks were selected to obtain an optimal representation of the entire tumor mass (i.e. blocks were selected that enabled maximal distances between selected tumor areas). One core from each selected block was then taken and placed in 10 different TMA blocks. This resulted in 10 different TMA blocks, each containing one tissue sample from each of our 189 selected patients. For subsequent mapping of molecular findings, the exact position from where each arrayed tumor sample had been retrieved was recorded in a database also containing all macroscopical images of our tumors. Each tissue sample was assigned to a defined tumor focus. The clinical and pathological features of our tumor cohort are provided in Table 1.

**Table 1:** Composition of the heterogeneity tissue micro array

	Study cohort on tissue microarray (*n* = 189)
**Age (years)**	
< 50	3
50–60	43
60–70	120
> 70	23
**Tumor stage (AJCC 2002)**	
pT2	127
pT3a	32
pT3b	29
pT4	1
**Gleason Score**	
≤ 3 + 3	4
3 + 4	144
4 + 3	33
≥ 4 + 4	8
**Lymph node metastasis**	
N0	130
N1	10
**Surgical margin**	
negative	160
positive	26

NOTE: Numbers do not always add up to 189 in the different categories because of cases with missing data. Abbreviation: AJCC, American Joint Committee on Cancer.

### Validation for presence of cancer on TMA spots

Technical issues represent a significant problem in studies analyzing heterogeneity because every false positive or false negative result will lead to a false classification as “heterogeneous”. Every effort was thus taken in this study to avoid false interpretations including immunohistochemical confirmation of presence of cancer for each sample. For this purpose, the antibody 34βE12 (clone MA903, Dako; 1:12.5; pH7.8) was used for basal cell detection and p504s (clone 13H4, Dako; 1:200; pH9.0) was utilized for AMACR detection. The EnVision™ Kit (DAKO, Glostrup) was used to visualize the immunostainings. For each tissue spot, presence or absence of normal prostate epithelium and the proportion of cancer tissue was recorded and quantified.

### Fluorescence *in-situ* hybridization

**Figure 4:**
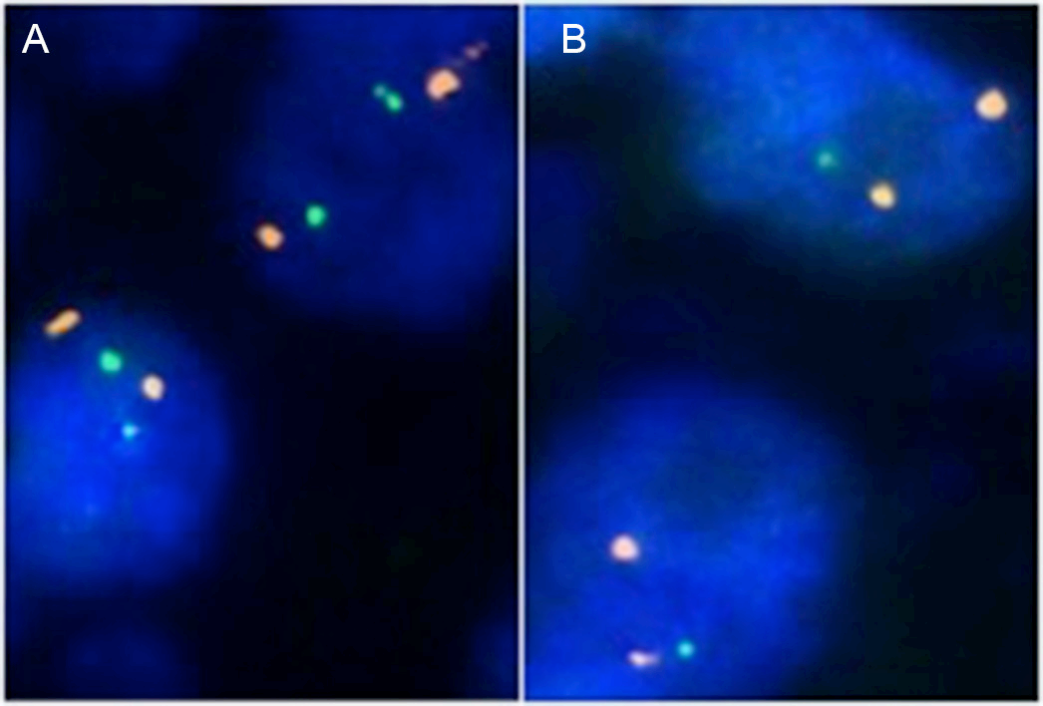
Examples of FISH findings showing (A) normal 6q15 signal numbers and (B) heterozygous 6q15 deletion Orange signals correspond to centromere 6, green signals correspond to the *MAP3K7* (6q15) gene locus.

A 4 μm TMA section was used for dual color fluorescence *in-situ* hybridization (FISH). Before hybridization, sections were deparaffinized and proteolytically pretreated with a commercial kit (paraffin pretreatment reagent kit; Abbott Molecular, Wiesbaden, Germany), followed by dehydration in 70%, 80% and 96% ethanol, air drying and denaturation for 10 min at 72°C in 70% formamide-2x SSC solution. For *MAP3K7* deletion analysis, a dual color FISH probe set consisting of two spectrum green-labeled BAC probes (BAC RP3–470J8, BAC P11–501P02; Imergenes, Germany) containing the *MAP3K7* gene and an adjacent up- and downstream region and one spectrum orange-labeled commercial centromere 6 probe (#06J36–06; Abbott), which was used as a reference. Hybridization was done overnight at 37°C in a humidified chamber, slides were washed and counterstained with 0.2 μmol/L 4′-6-diamidino-2-phenylindole in an antifade solution. For *MAP3K7* deletion analysis, each spot was evaluated and the predominant signal was recorded for each FISH probe. Tissue spots with lack of *MAP3K7* signals in all (tumor and normal cells) or lack of any normal cells as an internal control for successful hybridization of the *MAP3K7* probe were excluded from analysis. Heterozygous deletion of *MAP3K7* was defined as presence of fewer *MAP3K7* signals than centromere 6 probe signals of ≥ 60% tumor nuclei. Representative cases with and without *MAP3K7* deletion are shown in Figure 4.

### ERG immunohistochemistry

ERG immunohistochemistry data were available for each individual cancer spot from a previous study [1]. The antibody ERG (clone EPR3864, dilution 1:450, Epitomics) had been used for ERG protein detection in this study. Only nuclear ERG staining was considered. For each tumor sample the staining intensity was judged from negative to strong. Tumors with at least a weak ERG immunostaining were considered ERG-positive.

### Large section validation

To validate heterogeneity of *MAP3K7* deletion and *ERG*-fusion status, two cases with documented intrafocal heterogeneity and homogeneity for *ERG*-fusion status and intrafocal heterogeneity for *MAP3K7* deletion on our TMA were also subjected to large section analysis. In these cases, relevant cancer-containing tissue blocks (based on which TMA spots suggested heterogeneity or homogeneity) were cut and *MAP3K7* and ERG status were analyzed as described.

### Statistics

Statistical calculations were performed using JMP statistical software (SAS Institute, Cary, NC). Contingency tables were calculated with the chi^2^-test to investigate the relationship between the degrees of ERG heterogeneity.
